# Exploring Control Authority Preferences in Robotic Arm Assistance for Power Wheelchair Users

**DOI:** 10.3390/act13030104

**Published:** 2024-03-07

**Authors:** Breelyn Kane Styler, Wei Deng, Reid Simmons, Henny Admoni, Rory Cooper, Dan Ding

**Affiliations:** 1 Human Engineering Research Laboratories, VA Pittsburgh Healthcare System, Pittsburgh, PA 15206, USA; 2 The Robotics Institute, Carnegie Mellon University, 5000 Forbes Ave, Pittsburgh, PA 15213, USA; 3 Department of Rehabilitation Science and Technology, University of Pittsburgh, Pittsburgh, PA 15213, USA

**Keywords:** assistive technology, user agency, mixed method, activities of daily living, usability

## Abstract

This paper uses mixed methods to explore the preliminary design of control authority preferences for an Assistive Robotic Manipulator (ARM). To familiarize users with an intelligent robotic arm, we perform two kitchen task iterations: one with user-initiated software autonomy (predefined autonomous actions) and one with manual control. Then, we introduce a third scenario, enabling users to choose between manual control and system delegation throughout the task. Results showed that, while manually switching modes and controlling the arm via joystick had a higher mental workload, participants still preferred full joystick control. Thematic analysis indicates manual control offered greater freedom and sense of accomplishment. Participants reacted positively to the idea of an interactive assistive system. Users did not want to ask the system to only assist, by taking over for certain actions, but also asked for situational feedback (e.g., ‘How close am I (the gripper)?’, ‘Is the lid centered over the jug?’). This speaks to a future assistive system that ensures the user feels like they drive the system for the entirety of the task and provides action collaboration in addition to more granular situational awareness feedback.

## 1. Introduction

As software intelligence is incorporated more into actuated assistive devices, it is important to understand usability preferences for when and how people may be soliciting assistance from those systems. Wheelchair-mounted Assistive Robotic Arms (ARMs) have the potential to enhance the independence of users with upper-limb impairments [1–3]. As ARM ownership becomes more common, it is important to understand the user’s perspective on control and operation.

A trade-off emerges when incorporating software intelligence into assistive systems, forcing a choice between manual teleoperation and the ease offered by autonomous software assistance. Manual control offers advantages, as current robotic arms are controlled through the power wheelchair input, benefiting individuals skilled in operating power wheelchairs. Additionally, operating ARMs has been shown to increase user independence [3], which users value [4] despite inherent challenges such as longer operation times due to frequent mode switching. For example, an expert Kinova ARM user interviewed by Bhattacharjee et al. [5] noted that picking up a piece of fruit and bringing it to their mouth could take up to 45 min. Additionally, Herlant et al. [6] revealed that mode switching consumes 17.4% of task time, which increases cognitive load for users and highlights the mental demand [6,7]. However, these control challenges can be alleviated while preserving independence benefits through the integration of autonomous robotic arm functions [5].

Regarding software autonomy, robotic arm owners find this beneficial for repetitive or precise tasks [8], but where best to apply autonomous function and how it should adapt to best assist the user remain open questions [9–11], as, while autonomous robot software can increase task efficiency and reduce cognitive load [9], research has shown that full software autonomy is not always preferred when users have a choice [5,12,13]. This is a focal point of our study: investigating the balance of user control, a central theme in shared control paradigms.

A combination of manual and software assistance exists in shared control and shared autonomy platforms, which have been explored extensively—encompassing blended approaches [14–17], discrete switches [18,19], and mutual adaptation strategies [20] that augment manual control with autonomous functions—without investigating where in a task the user might prefer assistance most. Literature surveys distinguish between sharing paradigms, classifying shared control as autonomous functions initiated by the user and shared autonomy as autonomous functions initiated by the system (see [21]). Current research also shows that predicting a user’s intent and augmenting their control through shared autonomy is still a challenging area of research [22], as, while many studies focus on algorithmic implications or the robot’s ability to understand humans [23], there is a lack of direct insights derived from power wheelchair users who will incorporate ARM technology into their daily routines.

User preferences are important when evaluating the adoption of assistive technology with autonomous components [9]. This motivation often stems from the user’s desire to remain in control of such systems [24], a sentiment shared by robotic arm owners configuring autonomous tasks [8] and users of autonomous wheelchair navigation functions [25]. Our study directly investigates control authority preferences among potential future ARM users, specifically examining the choice between manual joystick operation and autonomous software control.

We compared two iterations of a kitchen task, gathering user feedback after introducing them to software autonomous control and manual joystick control. Initially, participants were introduced to a shared control system with predefined autonomous software actions (AutonSof v1.0.0). Subsequently, in the second phase, manual joystick control was introduced. In a final exploratory phase, users specified their preferred interaction with the system and where they desired robot assistance, termed interactive assistance.

This study yields the contributions listed below and outlines future design criteria for an interactive assistive manipulation system. The key takeaways include users’ preferences for autonomous assistance, contingent on the task and their personal state, and the users’ preference for driving the system at all times using the joystick. These insights stem from three investigative questions:

Are there parts of the task that users prefer to control with a joystick versus having autonomous robot control?

- Takeaway: Users exhibit a preference for manual control even in task segments characterized by lower success rates, longer task times, and increased mode switches (Section 3.4.1).
  When in a task does a user prefer increased assistance from the system?
- Takeaway: Users favor increased system assistance when experiencing fatigue, a need to enhance situational awareness, safety concerns, a requirement for greater precision, and, in certain instances, they choose not to utilize increased assistance at all (Section 3.3.1).
  What types of feedback or control does a user prefer to receive from the robot system during a manipulation task?
- Findings: This question lead to a set of design criteria (highlighted in Section 3.3.2) that recommend enhancing situational awareness, supporting multi-granular action selection, and incorporating multiple input methods when designing future assistive systems.

## 2. Methods

The study was conducted in Portland, Oregon, during the National Veteran Wheelchair Games (NVWG) in July 2023. NVWG is an inclusive community event open to all Veterans, emphasizing camaraderie and physical fitness over elite competition. It provides opportunities for Veterans with a range of mobility impairments to participate in adapted sports and recreational activities. Teams from different regions around the nation train with coaches, including recreational therapists; some participants are former Paralympic athletes. Participants for this study were recruited through flyers, word of mouth, and by possible participants approaching our recruitment table or assigned study room during breaks between their events. We tested 10 participants who use electric power wheelchairs and had no previous robotic arm experience.

### 2.1. Study Environment

**Figure 1.**
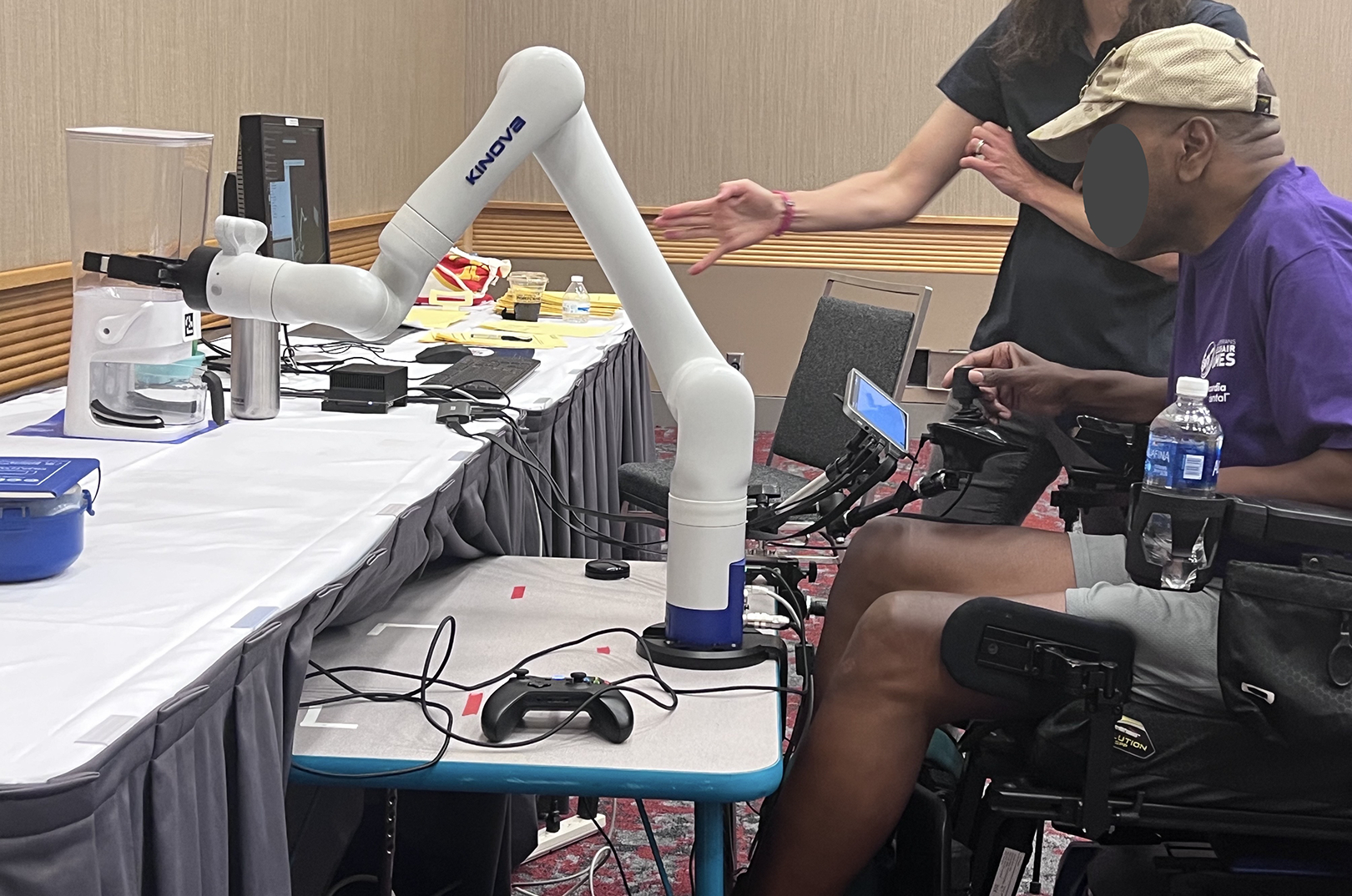
The study setup with robotic arm mounted on table in front of power wheelchair Veteran.

A Kinova Gen3 robotic arm was clamped to a table (Figure 1). All users positioned their wheelchair behind the robotic arm. A joystick and touchscreen, connected to quick-shift lever swivel clamps, were positioned on either side of their wheelchair, based on the participant’s dominant hand for joystick driving and dominant finger for touchscreen selection. The task setup was standardized for all participants. Each manipulation object’s position was reset to the same position and placed in the same order between each trial. Additionally, the robot arm always started in the default home position at the beginning of each trial.

Participants could switch between modes on the two-axis joystick by either pressing the top of the joystick or a larger button attached to it. The choice of button and its location depended on the upper extremity capabilities of each individual participant.

### 2.2. Participant Selection

Participants were screened for inclusion criteria: being over the age of 18, using a power wheelchair as their primary means of mobility, and self-reporting upper-limb impairment limiting their ability to perform daily activities. Participants were ineligible if they had impaired vision, difficulty carrying on a conversation due to impaired voice, or pressure ulcers that restricted sitting for more than three hours. We recruited 13 participants, but one was ineligible due to vision impairment, another did not exhibit upper-limb impairment, and a third did not attend their scheduled time, resulting in a total of 10 participants. One of the participants did not fully complete the second kitchen task operation scenario due to falling asleep, but returned later in the week, after resting, to complete the interview portion of the final interactive assistance scenario.

### 2.3. Study Procedure

The study was completed in no more than two hours and approved as a minimal risk study under United States Veteran Affairs IRBNet protocol number 1720292.

#### 2.3.1. Training

Participants went through the informed consent process and then filled out two surveys. The first was a demographic survey that included their diagnosis, wheelchair and ARM experience, personal care assistance, and perspectives on technology. The second survey was the Capabilities of Upper Extremity Questionnaire (CUE-Q) [26], a standardized self-assessment on upper-limb functionality. After the surveys, participants had a thirty-minute joystick control training session. During this training, we introduced the four joystick control modes: Translation X-Y, Translation Z/Wrist Rotation, Wrist Orientation, and Fingers. Participants were then instructed to touch the investigator’s hand, with the ARM gripper, at different locations before completing three training tasks. The first was retrieving and placing a water bottle (18 cm long) on the table, allowing practice of finger opening, closing, and translation modes. The second task was to grasp a thin light ring measuring 7.5 cm in length, 7.5 cm width, and 1.2 inches in depth, requiring wrist movement to achieve either an over-top or side grasp. The final task was grasping a thin notebook measuring 10 cm in width, 22.8 cm in length, and 0.6 cm in depth from the top of a small soup cup, emphasizing the Wrist Orientation mode. The position of all training objects was standardized between participants, ensuring consistency.

#### 2.3.2. Study Task

**Figure 2.**
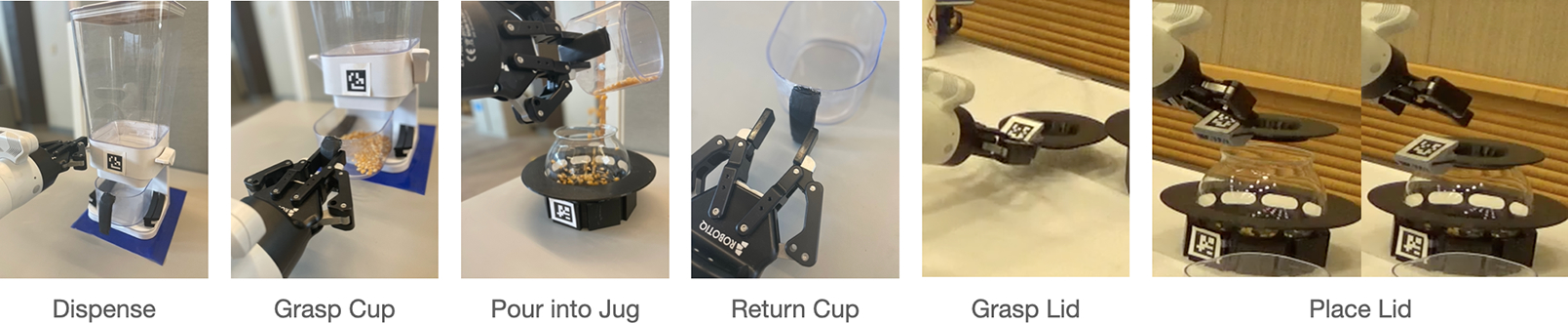
The six-step popcorn-making kitchen task.

After training, the user was introduced to a popcorn-making task, shown in Figure 2. The popcorn task comprised six steps: dispensing popcorn kernels into a cup, grasping the cup, pouring the kernels from the cup into a jug, returning the cup to the table, and finally, grasping the popcorn jug lid and placing it on the jug. They executed the task twice, and we recorded metrics on both occasions. The third iteration of the task was exploratory.

The user-initiated autonomous software mode (AutonSof) was always introduced first. Although, it is worth noting that the participants were introduced to manual joystick control first during training. Autonsof was selected for the initial trial to streamline the study duration and introduce the popcorn task as a training tool within this mode. This initial introduction of autonomous software functions aids in minimizing task time by illustrating arm movement strategies while providing task explanations. However, it is important to acknowledge the bias this may introduce, as discussed in Section 5.

**Figure 3.**
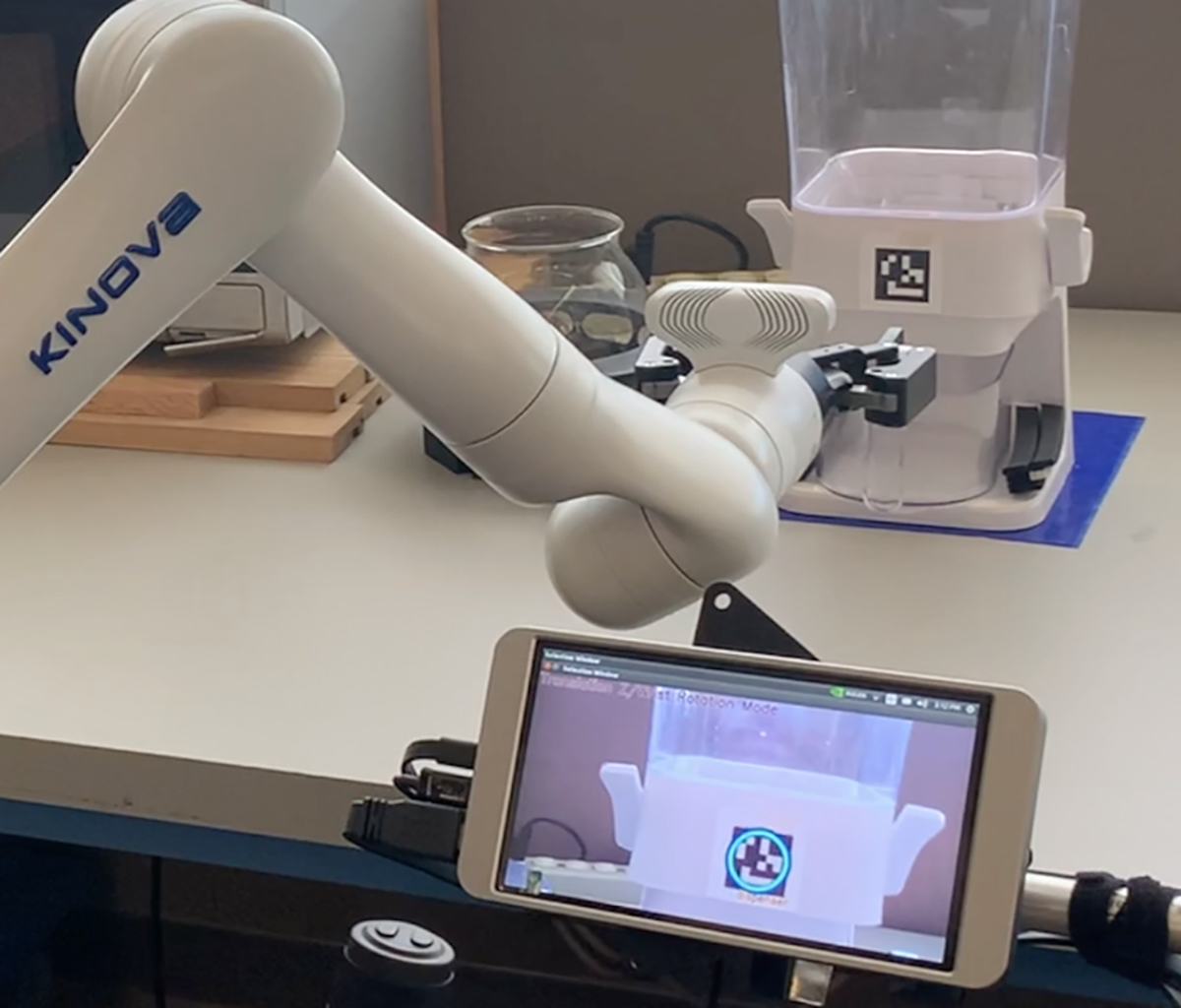
User-Initiated Software Autonomy: The dispenser tag is highlighted on the touchscreen, allowing the user to press a circle to transition to software autonomy.

In AutonSof mode, control alternates between the joystick and autonomous software, involving predefined autonomous actions as a shared control [18]. In this mode, the participant managed the robotic arm’s gross motions using the joystick. When the wrist-mounted camera aligned with fiducial tags in the environment, a circle was displayed on the screen for selection (Figure 3). The user then initiated software-controlled fine manipulation by touching the circle-highlighted fiducial tag on the touchscreen. The robotic arm automatically moved through direct Cartesian motion or motion-planned actions to achieve end-effector goals in Cartesian space. The controller utilized inverse kinematic solutions to drive the joints to the proper positions. The origin of the robotic arm’s kinematic chain is the center of the base, where it is mounted to the table in our experiments.

Table 1 provides an overview of the degree of software autonomy triggered by pressing the highlighted touchscreen tag. The first four popcorn steps are executed after pressing the dispenser tag, and the lid tag prompts the ARM to autonomously grasp and position the lid above the jug; control then returned to the user for lid placement on the jug.

During the second task iteration, the user manually performed all six steps using the joystick, disregarding the tag circles that appear on the touchscreen. The robotic arm’s end-effector was driven through joystick commands in Cartesian space.

After two iterations of the kitchen task, the user was instructed to perform the task for a third time during a final interactive assistance scenario. Users were provided with a script explaining the goal of this interactive assistance scenario: to gather perspectives on the preliminary design of a system capable of assisting at any point in a task, initiated by the user. Participants could switch control between joystick and autonomous arm movement at any time by instructing the investigator, who was always seated to the left of the participant and operated the robotic arm using an Xbox controller. Part of the script read by the investigator said, “I am acting as the software responsible for moving the ARM. Whenever you request assistance during a task, I will ask questions to determine where you are in the task and confirm which action I am assisting, then I will take over to help complete the current action you are performing”.

At the conclusion of the interactive assistance scenario, the user participated in a semi-structured exit interview, sharing overall perceptions. In addition to freely providing feedback during the third scenario, the following questions were asked:

1. What method of control did you prefer most and why (AutonSof, Manual, Interactive Assistance)?
2. If there are tasks you currently cannot do independently (without assistance), could you share your preferences regarding a robotic arm moving autonomously to assist, controlling the arm through a joystick, or receiving assistance from another person?
3. Can you provide some thoughts on how you prefer to give information and receive information from a robotic arm system?

### 2.4. Data Collection and Analysis

The survey study data encompassed demographic information, caregiver assistance, and user attitudes toward technology. Additionally, participants completed the 32-item self-assessment survey, CUE-Q [26], which evaluates upper extremity functionality, including arm, hand, and bilateral function. Each item is scored on a 5-point scale from 0 (unable/complete difficulty) to 4 (no difficulty), with a maximum score of 128.

While participants executed the task with AutonSof and manual joystick control, we recorded metrics on task success, completion time, and mode switches. Participants also filled out two standardized questionnaires: the NASA-TLX for workload assessment [27] and the System Usability Score (SUS) for system usability evaluation [28]. The NASA-TLX assesses workload along six dimensions: Mental Demand, Physical Demand, Temporal Demand, Performance, Effort, and Frustration. The SUS questionnaire consists of 10 items with Likert scale responses ranging from strongly agree to strongly disagree, and the SUS scale score ranges from 0 to 100, with higher scores indicating higher perceived usability. We conducted a Wilcoxon signed-rank test for each dimension of the NASA-TLX and for SUS scores to compare the two conditions (AutonSof and manual control) within subjects.

In the final interactive assistance scenario, we recorded instances when the user sought feedback from the system or initiated control authority transitions. We did not employ standardized questionnaires after this interactive assistance scenario since it did not necessitate a formal system evaluation, as the system included an investigator acting as the robot. Qualitative data from the semi-structured interview was transcribed and analyzed using thematic analysis [29]. Initially, the first two authors created a CodeBook of themes, and two participant transcripts were randomly selected and coded, with themes highlighted using NVivo software v14.23.0 (https://lumivero.com/products/nvivo/).The transcripts were thematically coded by three independent coders that then met to further clarify themes. After ensuring consistency among the three coders, we randomly selected two more participant interviews, which resulted in a convergence in themes. Remaining transcripts were then coded independently using this finalized CodeBook.

## 3. Results

### 3.1. Demographics

Table 2 shows demographic characteristics of the ten participants. Among them, eight were male, and two were female. Additionally, five of our participants identified as African American, while the remaining five identified as Caucasian. The duration of power wheelchair use ranged from 1 to 40 years, with a median ownership of 11 years. Furthermore, six of the participants self-reported having significant difficulty making decisions.

#### 3.1.1. Personal Care Assistance

Out of the ten participants, only one participant did not receive assistance from a personal care attendant.

Regarding personal care, seven participants indicated needing little to no assistance with feeding themselves, or transferring from their wheelchair to bed. Furthermore, eight reported being able to drink from a cup by themselves, and six responded that they could pour their own liquids.

#### 3.1.2. Technology Preferences

**Figure 4.**
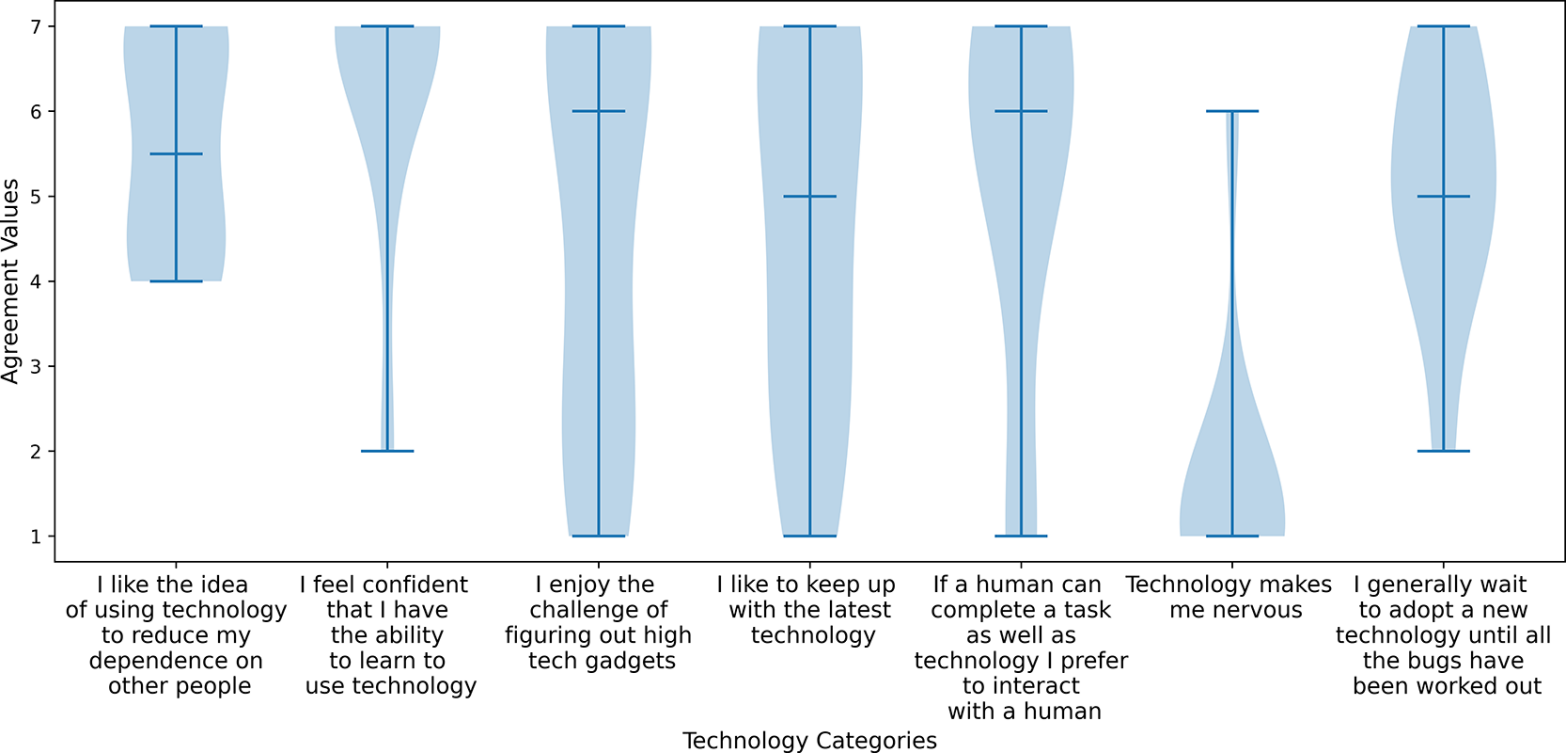
Participants attitudes towards technology.

Figure 4 presents the survey results, with participants ranking statements on the x-axis from 1 (strongly disagree) to 7 (strongly agree). Overall, nine participants felt confident in their ability to learn technology, with nine strongly disagreeing that it makes them nervous. However, only five agreed that they enjoy the challenge of figuring out high-tech gadgets, and like keeping up with the latest technology. More than half of the participants also agreed that they appreciate the idea of using technology to decrease dependence on others. Yet, eight participants prefer interacting with a person if it is possible to accomplish a task as effectively as with technology.

### 3.2. User-Initiated Autonomous Software and Manual Control Task Statistics

The following tables present completion times, mode switches, and success rates for both the AutonSof and manual control of the popcorn task. Table 3 displays the success rates for manual control, breaking down the task into its sub-components, with grasping the lid and placing the jug being the least successful. Table 3 also presents comparable success rates for AutonSof, indicating that the last two phases of the popcorn task were also the least successful, despite the overall higher success rates compared to the manual control condition. This improvement can likely be attributed to the robot’s more precise lid positioning. Table 4 provides task completion times and mode switch data for both the entire popcorn task and its individual subtasks. It is important to note that one participant did not complete the latter part of the manual task due to extreme exhaustion, resulting in missing task and mode switch times for the last four subtasks of that manual trial. The overall time is calculated for the manual task with the other nine participants, and the success rates reflect this lack of completion. One other participant that failed to manually place the lid, tried multiple times and then hit the circle on the touchscreen for autonomous assistance, which we marked as an incomplete trial. Their times were still included for the subtask and overall task. Additionally, during two out of the ten AutonSof trials, there were instances where the tag on the jug lid appeared blurry, causing delays in tag detection. In one case, the user had to manually grasp and place the lid without assistance from the software, requiring a retry, while in the other case, tag detection was delayed but still successful.

#### Task Workload and Usability

**Figure 5.**
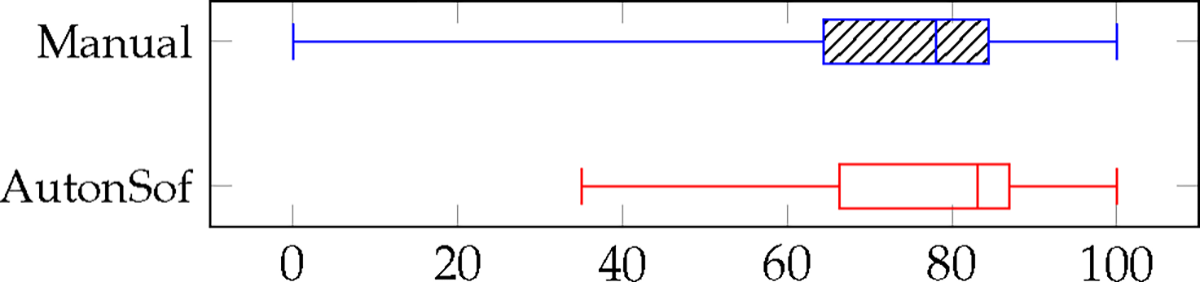
System usability scores for manual control (**top** boxplot) versus AutonSof control (**bottom** boxplot), both median values greater than 68.

**Figure 6.**
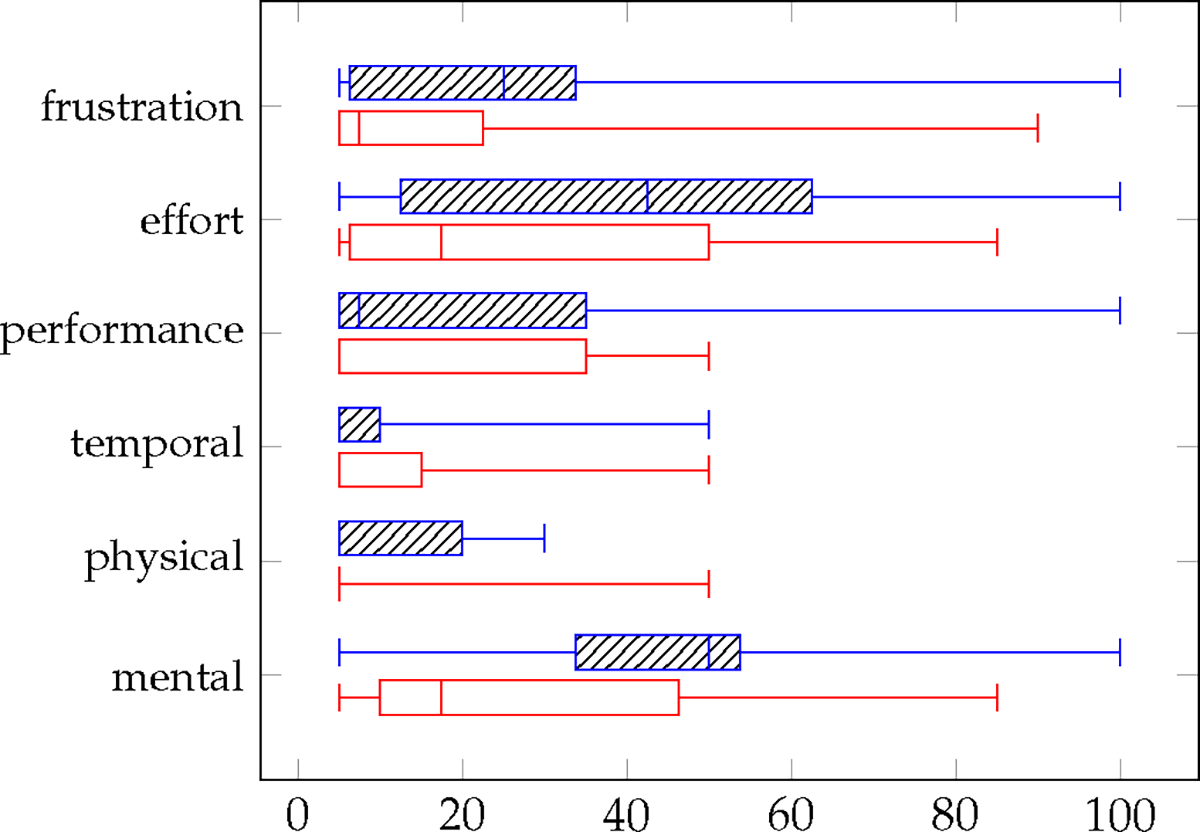
NASA TLX scores over six dimensions. Manual control shown as hashed line (**top** boxplot) and AutonSof control as open lines (**bottom** boxplot). Effort and mental demand higher for manual control.

The box plots presented in Figure 5 display the median, minimum, and maximum values for system usability scores, while Figure 6 provides the same statistics for the six workload dimensions of the NASA-TLX. To assess these metrics, we conducted a Wilcoxon signed-rank test for each dimension of the NASA-TLX, comparing the two control conditions (AutonSof and manual control) within subjects. Similarly, we performed this test to compare the same two controls for SUS scores. Notably, no significant difference was found in SUS scores. However, we did identify a significant difference in the mental demand dimension (*p* < 0.0078) for task workload.

### 3.3. Interactive Scenario Results

Out of the ten participants, seven asked the system something during the final interactive scenario. Among the seven who engaged with the system, all sought action assistance from the investigator; their reason for switching is in Section 3.3.1. Two participants asked for one-step commands like “close”, “up”, and “rotate”, while the remaining five requested more detailed instructions, such as “untangle arm”, “pour kernels”, and “get the lid and put it on the jug”. Interestingly, there was no specific subtask for which participants asked for more assistance than others. The requests were distributed across various task phases: two related to dispensing, two involving cup grasping, three for pouring kernels, and three for lid placement.

Two participants chose not to ask the system anything, and one did not complete the scenario. Four out of these seven participants also requested situational feedback, a design criteria highlighted in Section 3.3.2.

#### 3.3.1. Reason to Switch Control Authority

Three participants reported switching to robot autonomous control because they had difficulty seeing (either the arm was blocking their view or judging distances was challenging). A fourth participant said they tried a bunch and gave up, and a fifth said they “wanted to talk to the robot and got tired at the end”. The sixth participant, wanted assistance with untangling the robot (“I got the arm crossed too close on itself”), citing future safety concerns as reasons for further collaboration, such as avoiding getting too close to the stove or needing the robot to handle hot beverages more automatically. The seventh participant preferred robot assistance for precision and to avoid wasting food, specifically avoiding kernel spills.

#### 3.3.2. Interactive Assistance System Design Criteria

**Figure 7.**
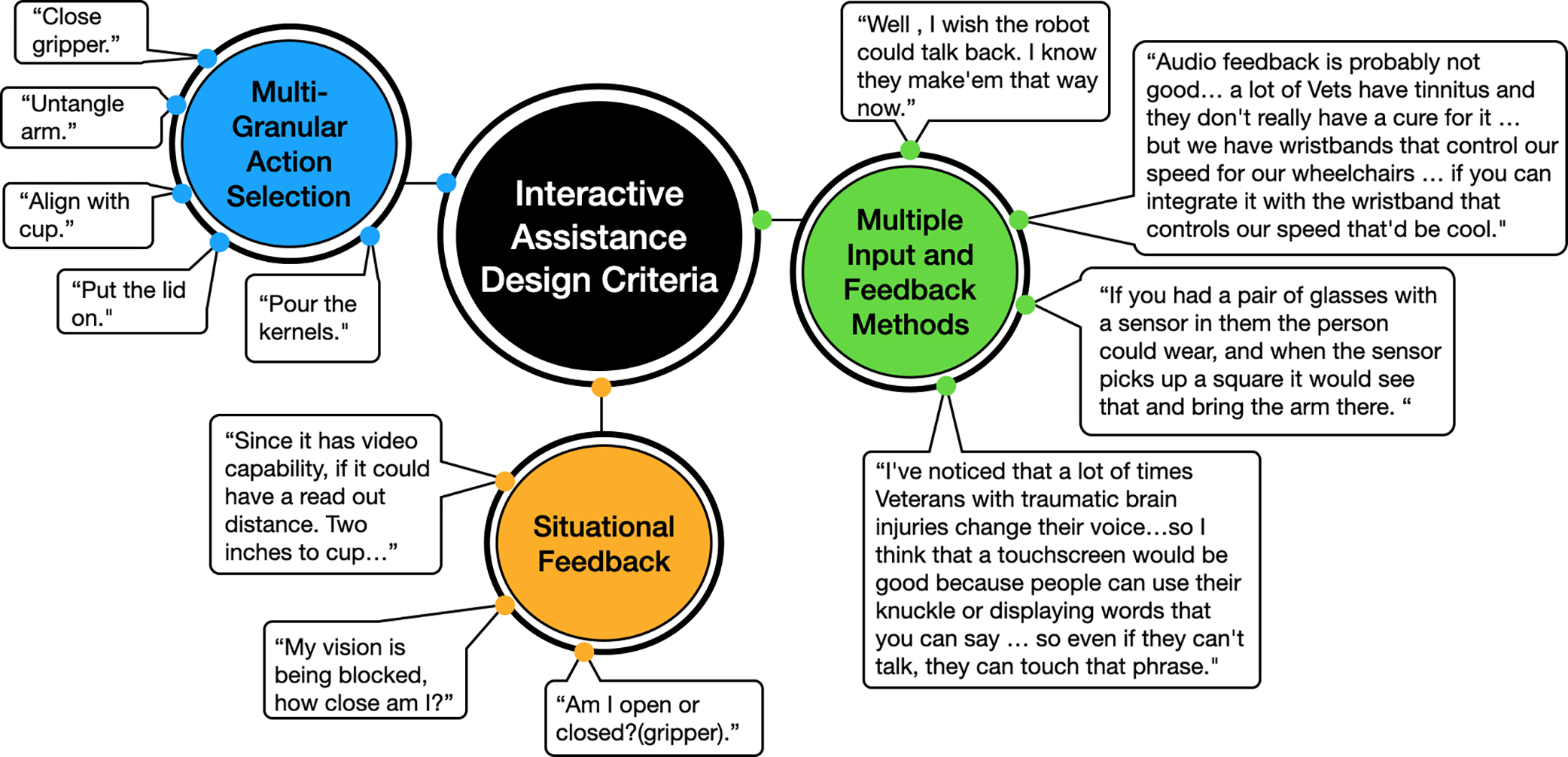
Diagram with three main design criteria for an Interactive Assistance System.

Figure 7 shows three main design criteria that were suggested by participants to enhance a future interactive assistance system. These include better situational feedback to the user, the option of having a multi-granular action assistance selection (as users often jumped between specific and more general actions if requesting assistance), and finally the ability to interact with the system in multiple ways. Quotes support each of these design criteria.

For situational awareness, users often struggled with depth perception and alignment between objects. Many asked for feedback on whether their grasp would be successful before closing the gripper, or if their popcorn cup was properly centered over the jug before starting to deposit kernels. While many of the feedback requests were visual, users also struggled to realize when the hand or the popcorn cup might brush up against other objects, altering their preconceived idea of the interaction.

For multi-granular action assistance, users had different approaches. Some gave only one-step commands, while others asked for assistance throughout the task but changed the commanding action based on how much of the task was left or if they had tried multiple times and wanted more encompassing assistance.

Participants also shared interaction method preferences for both conveying information to the system and receiving feedback. Participants suggested both a voice-activated interface with the option of touch, acknowledging that voice control may not work for everyone. They also suggested visual indicators for feedback on the environment state or a wrist haptic feedback device. These interaction methods would need to complement manual control, as most participants preferred it, as described in Section 3.4.1. However, overall, there was a wide variety of suggestions depending on the user’s abilities and preferences.

### 3.4. Interview Themes

During the exit interview, all participants agreed that training was sufficient. One participant praised the training, saying, “No, it was good, standard procedure for training. You gave better training than most Sergeants give in the military”. Additionally, we inquired about their control preferences and the reasons behind them, which led to the identification of the themes related to control authority preferences and participant mentalities presented in Table 5. We will now delve into the subthemes that emerged from these overarching themes.

#### 3.4.1. Participants Prefer Joystick Control for Its Sense of Independence, Control, and Accomplishment

Users expressed a strong desire to do things by themselves. They discussed wanting to feel like they were driving the system at all times. One user said:

I like using, you know myself . . . especially once I got to be familiar I feel more accomplished in that, it makes me feel more independent, that I’m actually doing it. . . . It’s not my hand doing it, but it is that my hand is on the joystick.

Another participant told a story of what their father taught them, which illustrates their preference for joystick mode.

My father always taught me that there is nothing like hands on. All these different gadgets and stuff they are fine and dandy, but nothing beats hands on. You’ve got to get your hands in the dirt . . . it makes you feel better once you finish something and you say, “I do this with my hands”, and (when) the machine, they do it then you have not done nothing but sit down.

#### 3.4.2. Participants Had Positive Impression of Robotic Arm Moving Itself

None of the participants were intimidated when the robotic arm moved itself. They were generally impressed with the robot’s autonomous capabilities. Additionally, one person even commented that the arm moving on its own helped them better understand the task. Below are a couple of quotes showcasing users’ positive impressions.

Having a robot complete the task is cool as heck. It would save time . . . and the frustration level would drop.

I think it’s amazing. I did not know that such devices existed, so I think it’s brilliant.

One user also joked that:

The robot was pretty good. It did not take them longer than I did. . . . it does not ask for tips or a raise.

#### 3.4.3. Participants Like Robot Automatically Moving as a Backup Option

Users provided examples where they liked the idea of the robot assisting them as an alternative to the joystick control, but they did not want it engaged the entire time. For example, they recognized that it could be useful on days they were feeling off and reassuring knowing something could assist if they got stuck.

I like the second one (manual control). Because I like to be more independent, but if I could not move that well or got sick, I would use the computer.

The interactive control was probably the nicest one to use because when I got stuck . . . it is nice to know . . . if I need help, the computer can help me, but I also knew I could fix it if I had to because I did it before.

#### 3.4.4. Participants Have an Independent Mindset

Overall, participants were highly independent, as illustrated by their quotes. Many had stories of the importance of having a proactive attitude in their own lives; otherwise, they might not have achieved success.

I’m not a coward. (response when told could say “robot takeover”)

With Spinal Cord (injury), we do not like to say we cannot do it. The ones who do, they got problems.

How much are you going to be dependent on yourself? So you do not need to say, “okay, robot takeover” because . . . We are a type of people, whereas we do not want nobody to be pourin over us. Yes, it’s unfortunate us being here. . . . However, the bottom line is we are here.

No, no the software could not have done it better than me.

This also includes the desire to be in control as reflected in the following quotes:

Nobody wants to feel that you have to always have somebody to do things for you.

I just prefer to be in control of stuff and not having all mechanical assistance.

I would prefer a person to teach it to me . . . not do it for me. Let me do it, but just show me how to do it.

#### 3.4.5. Participants Have a Learning Mindset

Many preferred to learn by doing. This was another reason given for their preference towards joystick control. Many expressed how they liked to initially figure things out on their own and they felt they understood the system better by manually controlling it.

I am a kind of person who likes to learn things and I want to learn to do it. . . . When it comes to iPhones, a lot of people my age are afraid of technology and I’m not. I want to learn and I want to know. I want to know how does is it work? How do I do this? How do I do that? So that is the way my brain works.

I like having the computer assist, but that is going to require, in my mind, a lot of programming on standard tasks so I guess my favorite would be freehand . . . because I can learn how to play with it myself, the more you play with it, the easier it becomes, the more . . . expert you become with it.

## 4. Discussion

While most of the surveyed participants expressed confidence in their ability to learn technology, they did not demonstrate a strong inclination to embrace technology before potential issues were resolved, nor did they prioritize technology over human assistance (see Figure 4). Their attitudes towards keeping up with and mastering the latest tech were also mixed, suggesting that this population may be less tech-savvy. Additionally, our participant group consisted mainly of older power wheelchair users, whom exhibited a strong desire for self-sufficiency and generally did not have severe upper body impairments, as indicated by their CUE-Q scores. However, it is worth noting that the CUE-Q questionnaire does not account for factors such as loss of sensation, spasticity, or touch. For example, one participant with a CUE-Q score of 128 had no feeling in their hands, which makes precise joystick proportional control difficult.

When comparing the performance of AutonSof and manual controls, it was evident that manual control trials took longer and required more mode switches, as expected. Manual control trials also exhibited a much larger standard deviation over completion times due to the increased number of subtask re-tries, whereas AutonSof showed greater consistency in completion times. Subtask completion times were longest for lid grasping and placement, which can be attributed to two factors; first, these subtasks involved numerous wrist movements, and second, participants found it challenging to ensure that the lid was correctly centered over the jug before releasing it. Users also noted that the arm’s position on the table sometimes obstructed their field of view, contributing to these difficulties. Although a wheelchair-mounted ARM would typically be positioned on the side of the wheelchair, concerns about depth perception and situational awareness would persist, especially among users with limited trunk control who predominantly remained in a seated position. These concerns were raised by four out of the seven participants who interacted with the system during the interactive assistance scenario.

Regarding mental workload, both effort and mental demand were higher for manual control compared to AutonSof control, with mental demand being the only workload dimension that showed a statistically significant difference. This can be attributed to the substantial mode switching required during 2-axis joystick operation.

In terms of system usability, both AutonSof and manual control tasks achieved *good* SUS scores, surpassing the 68 cutoff [28]. However, one participant had negative impressions of the usability of both systems, creating outliers possibly due to exhaustion and mental fatigue. Nevertheless, this situation highlights the potential of adding software autonomy, enabling users to complete tasks they might otherwise be unable to due to health conditions or fatigue.

Despite the advantages of AutonSof control, including efficiency, reduced cognitive load, and the ability to complete tasks while fatigued, seven out of ten participants preferred manual joystick control, while all participants had a positive impression of the robotic arm’s autonomous capabilities, when asked to choose their preferred control scenario among AutonSof, manual, or interactive, three preferred the interactive assistance control, while the remaining seven favored the joystick. This leads to our first research question, Are there parts of the task that users prefer to control with a joystick versus having autonomous robot control?, answered as an overall desire for manual control illustrated in Section 3.4.1. This preference may also be attributed to the self-sufficient attitude highlighted in Section 3.4.4. For example, one participant shared how he avoids getting frustrated because, without a positive mindset and self-reliant attitude, he believes he would have not “lasted very long in this chair”. Therefore, it is also important how information is conveyed to the user from these systems. If the language used for assistance is equivocal to *help* or having it *takeover* it could negatively impact usage.

Additionally, even though participants express a preference for manual control when directly ranking the methods, Section 3.4.3 reveals that users still acknowledge the benefits of autonomous software control. They discuss it as a backup option, though this might be because they did not fully comprehend its potential for use in conjunction with the joystick, rather than as an entirely alternative mode.

This leads to a less straightforward answer of our next research question: When in a task does a user prefer increased assistance from the system? According to Section 3.3, there was no specific stage in the task where all users uniformly preferred additional assistance. Section 3.3.1 also highlights the various reasons seven of the ten participants chose to get assistance, including better situational feedback, feeling tired, personal safety concerns, or the need for higher precision. In some instances, users declined assistance even when certain parts of the task were progressing slowly. Perhaps, this is attributed to the user’s perceptions of what constitutes getting assistance or the fact that these were novice users focused on the immediate task, driven by a motivation to complete it independently within a limited time frame.

Therefore, further research is necessary, especially with systems deployed for more extended periods, to better understand assistance preference. Examining the potential benefits of user-initiated software autonomy may reveal instances where novelty wears off, and such software could significantly save time, especially for frequently performed tasks. This was suggested in a previous study involving robotic arm owners who preferred autonomous modes for repetitive tasks [8].

Finally, we offer design insights regarding the question, What types of feedback or control does a user prefer to receive from the robot system during a manipulation task?, as described in Section 3.3.2. Users indicated a preference for situational feedback regarding gripper positions in the environment, the ability to issue both one-step and multi-step assistance commands, and suggested options for multiple input methods. Therefore, future areas of research include how best to present situational feedback to the user as they are driving the system while minimizing distractions, or information overload. Additionally, users asked for assistance with diverse actions, prompting further investigation into how action selection changes within the task, and based on user interaction with different objects. This presentation of actions may also be personalized, with users desiring assistance for completion of larger subtasks, or more atomic one-step commands—something that is learned as the user interacts with the system.

Moreover, there is a need for a more in-depth exploration of how to effectively convey information to and from the system. Given the multitude of suggestions for feedback and system interaction, coupled with the varying preferences and abilities of users, it is important to design this in a modular way that can adapt and meet the specific needs of the intended user.

## 5. Limitations

Our study has two primary limitations: population bias and limited study time. Population bias is evident because the NVWG attracts a competitive, outgoing, and socially engaged group of attendees. Additionally, our participants demonstrated better upper-limb function compared to current ARM owners, as indicated by higher CUE-Q scores (current owners have a median value of 5 and an average of 14) [30] and self-reported ability to perform tasks such as drinking and pouring liquids (Section 3.1). However, one participant said, “my friend who has a higher level of injury might benefit more from using the autonomous control”.

The second limitation relates to the constrained study time due to the NVWG schedule. This limitation impacts the number of trials we could conduct and may not fully reflect how owners would use the system in their daily lives, where they would be more proficient and familiar with controls during various tasks, without the time constraints imposed by the Games. Furthermore, the study order effect (conducted to save time) could have influenced user preferences for manual control. Counterbalancing the sequence of testing user-initiated software autonomy and manual control might have provided more balanced results. Additionally, novice users may have difficulty envisioning the long-term benefits of software autonomy, which could become evident with constant use over time, highlighting the importance of engaging users in longer-term trials.

Lastly, we recognize the bias manual control introduces. Current commercial assistive robotic arms operate using the same input as is used to drive the power wheelchair, such as 2D-joystick, head array, or sip and puff; most commonly a 2D-joystick. This introduces frequent mode switching due to operating a high degree-of-freedom robotic arm with a low degree-of-freedom input control. This also limits the arm’s ability to operate in more than two directional movements at a time (i.e., a curve), increasing the potential for error. Therefore, longer execution time and increased mode switching is anticipated in manual mode. However, manual mode was still chosen as a comparison. This decision was made to investigate user perspectives related to their control initiation preference between the factory default and a more intelligent software system and gather design feedback for future iterations.

## 6. Conclusions

In conclusion, our study explored control authority preferences among future ARM users. Through a mixed-methods investigation of user perspectives, we provide valuable insights for the design of future collaborative ARM systems that prioritize user autonomy and control. Several key design considerations emerge from our findings. Firstly, the development of an interactive feedback system for assistive technology, potentially leveraging advanced models, could tailor to a user’s ability and usage style. Secondly, an interactive assistive system should offer variable granularity in action selection, allowing users to choose single- or higher-level actions, and adapt the state feedback based on task progression, with options for voice or touchscreen commands. Lastly, the user’s sense of control must be maintained at all times. If interactive components can seamlessly integrate with the user’s physical control input in a cognitively intuitive manner, this would be the ultimate goal.

## Figures and Tables

**Table 1.** Discrete User-Initiated Autonomous Software.

Tag Placement	Joystick Control	Autonomous Control
Dispenser	Move robot to dispenser tag.	Dispense popcorn, grasp cup. Pour kernels in jug.
Popcorn Lid	Move robot to lid tag. Wait, then, release lid.	Grasp lid, place above jug.

**Table 2.** Demographic information and Capabilities of Upper Extremity Questionnaire functional score.

Diagnosis	Age	CUE-Q (Max 128)

Spinal Cord Injury (SCI)	76	115
Stroke	62	85
SCI	63	81
SCI	66	11
SCI	67	79
Multiple Sclerosis (MS), relapsing remitting	40	128
MS, secondary progressive	57	118
SCI	57	102
Stroke, hemiplegia	67	68
Stroke	61	64

**Table 3.** Manual Control and AutonSof Task Success Rates.

	Manual Control			Autonomous Software		

	Success First Try	Success with Retries	Incomplete	Success First Try	Success with Retries	Incomplete

Dispense	90%	10%	0%	100%	0%	0%
Grasp Cup	80%	20%	0%	100%	0%	0%
Pour into Jug	80%	10%	10%	100%	0%	0%
Return cup	90%	0%	10%	100%	0%	0%
Grasp Lid	30%	60%	10%	90%	10%	0%
Place Lid	30%	50%	20%	90%	10%	0%

**Table 4.** Task Completion Time and Mode Switch Statistics.

	Task Time (s) AutonSof	Task Time (s) Manual	# Mode Switch AutonSof	# Mode Switch Manual

Dispense	63.1 ± 39.5	137.9 ± 93.4	4.7 ± 5.1	18.6 ± 11.7
Grasp Cup	16.2 ± 2.3	129.3 ± 97.4	0 ± 0	18.1 ± 9.0
Pour into Jug	32.3 ± 7.7	177.3 ± 71.0	0 ± 0	23.3 ± 15.5
Return cup	27.3 ± 20.5	72.5 ± 35.2	0 ± 0	16.2 ± 2.3
Grasp Lid	117.8 ± 54.1	224.5 ± 73.8	6 ± 6.4	12.7 ± 11.0
Place Lid	71.3 ± 29.8	332.5 ± 335.5	6.4 ± 5.7	45.9 ± 31.4

Overall Values	328.1 ± 79.3	940.4 ± 236.1	17.1 ± 10.1	164.6 ± 85.8

**Table 5.** Themes and Subthemes for Interactive Assistance System Preferences.

Themes	Subthemes

Control Authority Preferences	—Participants prefer joystick control for its sense of independence, control, and accomplishment; —Participants have positive impression of robot automatically moving; —Participants suggest software autonomy as a backup option.

Mentalities	—Participants have independent mindset; —Participants have learning mindset.

## Data Availability

The data presented in this study are available on request from the corresponding author. The data are not publicly available due to being restored by the US Department of Veterans Affairs, and are subject to the approval of the relevant authority.

## Abbreviations

The following abbreviations are used in this manuscript:

ARM: Assistive Robotic Manipulator
AutonSof: Autonomous Software Control
NVWG: National Veteran Wheelchair Games
CUE-Q: Capabilities of Upper Extremity Questionnaire
SUS: System Usability Survey
